# Silicon amendment is involved in the induction of plant defense responses to a phloem feeder

**DOI:** 10.1038/s41598-017-04571-2

**Published:** 2017-06-26

**Authors:** Lang Yang, Yongqiang Han, Pei Li, Fei Li, Shahbaz Ali, Maolin Hou

**Affiliations:** 1 State Key Laboratory for Biology of Plant Diseases and Insect Pests, Institute of Plant Protection, Chinese Academy of Agricultural Sciences, Beijing, 100193 China; 2 Southern Regional Collaborative Innovation Center for Grain and Oil Crops in China, Changsha, 410128 China; 3 Institute of Plant Protection of Hunan Province, Changsha, 410125 China

## Abstract

Plant resistance to herbivores is a key component in integrated pest management. In most cases, silicon (Si) amendment to plants enhances resistance to herbivorous insects. The increase of plant physical barrier and altered insect behaviors are proposed as mechanisms for the enhanced resistance in Si-amended plants, but our understanding of the induced mechanisms involved in Si-enhanced plant resistance to phloem-feeding insects remains unclear. Here, we show that Si amendment to rice (*Oryza sativa*) plants impacts multiple plant defense responses induced by a phloem-feeder, the brown planthopper (*Nilaparvata lugens*, BPH). Si amendment improved silicification of leaf sheaths that BPH feed on. Si addition suppressed the increase of malondialdehyde concentration while encouraged increase of H_2_O_2_ concentration in plants attacked by BPH. Higher activities of catalase and superoxide dismutase were recorded in Si-amended than in non-amended BPH-infested plants. BPH infestation activated synthases for secondary metabolites, polyphenol oxidase and pheny-lalanine ammonia-lyase, and β-1,3-glucanase, but the activation was greater in Si-amended than in non-amended plants. Taken together, our findings demonstrate that Si amendment interacts with BPH infestation in the induction of plant defense responses and consequently, to confer enhanced rice plant resistance.

## Introduction

The brown planthopper (*Nilaparvata lugens* Stål; BPH) is a destructive and migratory insect pest that feeds on the leaf sheath of rice (*Oryza sativa* L.). It damages plants by ingesting phloem sap via its piercing mouthparts, which generally results in a symptom of hopperburn in susceptible plants when pest populations are large. BPH also damage rice plants by acting as vector of several rice viruses. In the last one or two decades, BPH caused frequent heavy damage to rice crops^1^. Although traditional chemical pesticides can afford substantial control of BPH, it is reported that BPH has developed resistance to several chemical insecticides^2^. Long-term application and misuse of chemical insecticides kill natural enemies that help maintain BPH population in check, resulting in resurgence of the pest. Additionally, chemical pesticides can pollute water and soil, and residual concentrations on crops can also damage human health. Therefore, there is an urgent need to develop effective and ecologically sound alternative methods to improve the pest control. Silicon (Si) amendment may be one of such potential alternatives^3^.

It has been demonstrated that Si amendment to plants can afford substantial protection from herbivorous damage by enhancing plant resistance^4–8^. Si amendment enhances plant resistance to herbivores through constitutive defense and/or induced defense. Enhanced constitutive defense is believed to be a result of increased rigidity and reduced digestibility of plant tissues due to additional amorphous silica deposition in Si amended plants^6, 9, 10^. Si-mediated resistance may also be realized through priming chemical defense in plants^5, 11, 12^ and augmented release of herbivore-induced plant volatiles that attract natural enemies of the attacking pests^13^.

Herbivorous feeding usually induces a battery of chemical defense responses in plants, on which Si is reported to play a role. As one of the physiological responses to herbivory^14^, malondialdehyde (MDA) usually experiences increase in concentration and has been extensively used as a biomarker of the degree of cell membrane damage^15^. The rapid and transient production of reactive oxygen species (ROS) by the plant, particularly H_2_O_2_, in the early phase of plant responses to biotic stress activates an array of plant defense mechanisms^16^. Antioxidant enzymes, superoxide dismutase (SOD), catalase (CAT) and peroxidase (POD), which are important in maintaining a balance of ROS^17^, are shown to be activated more in Si-amended than in non-amended infested plants^12, 18^. Polyphenol oxidase (PPO) and pheny-lalanine ammonia-lyase (PAL), catalyzing the synthesis of herbivore resistant secondary metabolites (lignin and phenols), show changes in activities similar to the antioxidant enzymes in response to Si amendment and herbivorous attack^5, 12^, although recent reports indicate a positive relationship between root herbivore performance and root phenolic concentrations^19, 20^. Plant β-1,3-glucanase is hydrolytic enzyme and located either in the vacuole or secreted into extracellular spaces. Acting alone and particularly in combination with chitinase, β-1,3-glucanase contributes to plant defense^21, 22^.

For BPH, it has been proved that rice lines treated hydroponically with high Si concentrations reduce performance of BPH^7^. We further determined that Si amendment decreased BPH population growth through impairment of feeding behaviors and reduced feeding amount in BPH^8^. However, the physiological mechanism underlying Si-mediated feeding impairment and decreased population growth remains unknown.

The objective of this study was to explore defense responses in Si-amended rice plants to BPH infestation in an attempt to elucidate the physiological mechanisms for the enhanced plant resistance associated with Si amendment. Silicification of rice leaf sheaths and concentrations of MDA and H_2_O_2_ were measured for their responses to Si addition and BPH infestation. Activities of antioxidant enzymes, synthases of secondary metabolites, and β-1,3-glucanase were determined in plants amended with Si and infested by BPH or not. The results of these measurements were linked to the enhanced resistance to BPH in Si-amended plants.

## Results

### Plant silicification in response to Si amendment and BPH infestation

As showed by scanning electron micrographs, the dumbbell-shaped silica cells distribute in rows along the veins (Supplemental Fig. S1). In plants attacked by BPH, row of silica cells per 1 mm^2^, number of silica cells per 1 mm row, and area of silica cells in Si-amended (+Si) plants were 14.3%, 2.6% and 5% higher than those in Si-non-amended (−Si) plants, respectively, while length and width of silica cells were not different between +Si and −Si plants (Table 1). In un-infested plants, values of each of the histological parameters of silica cells were higher in +Si plants than in −Si plants (Table 1). Si content in leaf sheaths of +Si plants was 10-folds of that in −Si plants (Supplemental Fig. S2). It is evident that Si amendment has improved silicification of leaf sheath.

**Table 1:** Effects of Si amendment and *Nilaparvata lugens* infestation on silicification of rice leaf sheaths.

Treatments	Rows of silica cells per 1 mm^2^	No. silica cells per 1-mm row	Area of silica cells (µm^2^)	Length of silica cells (µm)	Width of silica cells (µm)
−Si−BPH	7.6 ± 0.16 a	40.4 ± 0.25 ab	236.6 ± 3.82 a	19.9 ± 0.23 a	17.4 ± 0.22 a
−Si + BPH	7.9 ± 0.07 a	39.8 ± 0.32 a	240.4 ± 3.01 ab	20.5 ± 0.22 ab	18.1 ± 0.22 b
+ Si−BPH	8.7 ± 0.23 b	41.7 ± 0.14 c	268.6 ± 3.94 c	20.8 ± 0.22 b	19.0 ± 0.20 c
+ Si + BPH	9.0 ± 0.25 b	40.8 ± 0.19 b	251.7 ± 2.77 bc	21.5 ± 0.15 bc	18.5 ± 0.16 bc
n	15	75	100	100	100

+Si = silicon amendment at 112 mg Si/L nutrient solution to rice plants, −Si = no silicon amendment. +BPH = infestation by *N. lugens*, −BPH = no infestation. Values are means ± SE (n, number of biological replicates). Different letters following the means in the same column denote significant difference at *P* < 0.05 via Tukey’s multiple range tests.

### Responses of plant MDA and H_2_O_2_ contents to Si amendment and BPH infestation

**Figure 1:**
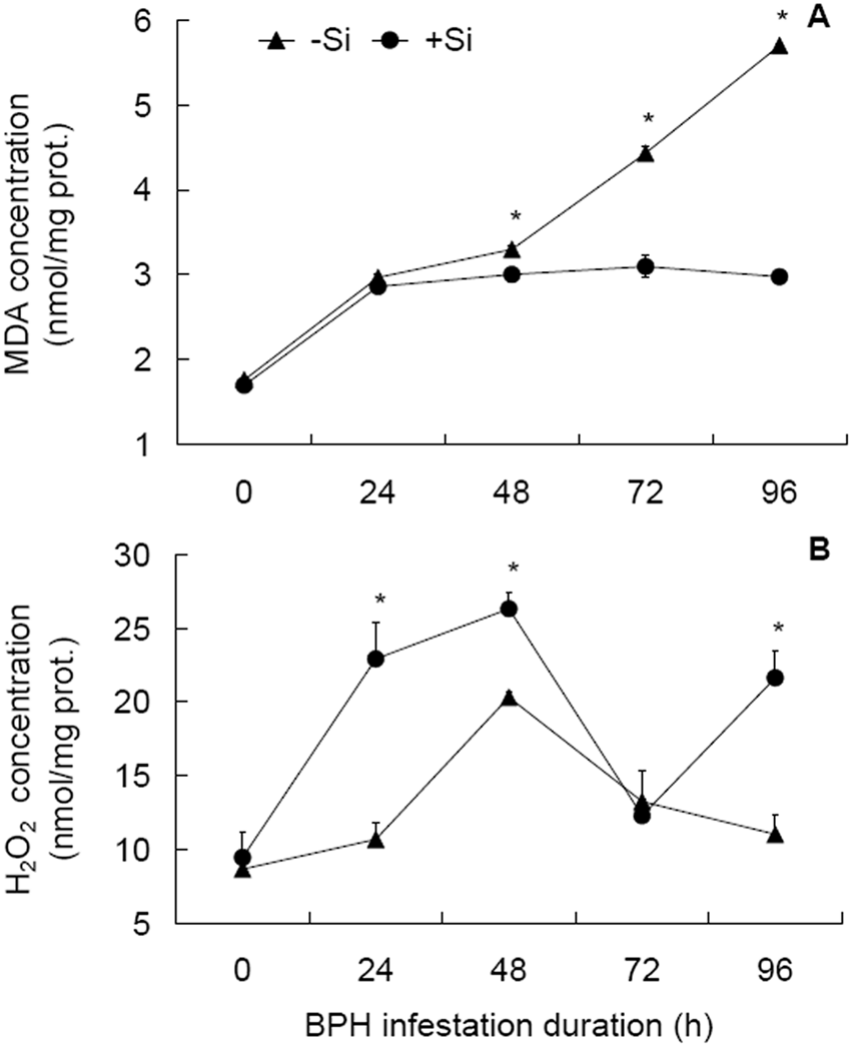
Concentrations of malondialdehyde and H_2_O_2_ in rice leaf sheaths in response to Si amendment and BPH infestation. (**A**) Malondialdehyde, MDA. (**B**) H_2_O_2_ + Si = silicon amendment to rice plants at 112 mg Si/kg nutrient solution, −Si = no silicon amendment. Error bars represent 1 × SE. *n* = 3 (biological replicates). *Significant difference between + Si and −Si plants at a certain time post BPH infestation at *P* < 0.05 via independent samples T test.

MDA is one of the physiological index of plants under stress^14^. ANOVA showed that Si treatment, BPH infestation duration and their interaction all significantly influenced MDA concentration (Table 2). MDA concentrations in both the +Si and −Si plants responded positively to BPH infestation, as indicated by a significant (*t* ≥ 11.014, df = 4, *P* ≤ 0.001) increase at 24 hpi (Fig. 1A). Thereafter, in + Si plants, MDA concentrations maintained at a relatively flat level while in −Si plants, increased significantly at 72 from 48 hpi and at 96 from 72 hpi (*t* ≥ 11.466, df = 4, *P* ≤ 0.001). Between the Si treatments, MDA concentrations were higher in −Si plants than in +Si plants at 48, 72 and 96 hpi by 9.9%, 43.1% and 91.5%, respectively. It is obvious that Si amendment inhibited the increase of MDA concentration in response to BPH infestation.

**Table 2:** Two-way analysis of variance for significance (*P* value) of the effects of Si amendment and *Nilaparvata lugens* infestation duration on rice physiological parameters.

Treatment	MDA^a^	H_2_O_2_^a^	Soluble Protein^a^	CAT^b^	SOD^b^	POD^b^	PPO^b^	PAL^b^	β-1,3- glucanase^b^
Si amendment (A)	<0.001	<0.001	0.041	<0.001	<0.001	<0.001	0.032	<0.001	0.003
BPH infestation duration (B)	<0.001	<0.001	0.002	<0.001	0.053	0.156	<0.001	<0.001	<0.001
A × B	<0.001	0.001	0.001	<0.001	0.018	0.152	<0.001	0.002	0.058

^a^Concentrations measured, ^b^Activities measured. Si amendment at 0 or 112 mg Si/kg nutrient solution, *N. lugens* (BPH) infestation duration: 0, 24, 48, 72 or 96 h.

H_2_O_2_ functions as a threshold trigger for hypersensitive cell death and as a diffusible signal for induction of cellular protectant genes in surrounding cells^14^. Like MDA, Si treatment, BPH infestation duration and their interaction all significantly influenced H_2_O_2_ concentration (Table 2). H_2_O_2_ concentrations also responded positively to BPH attack in both +Si and −Si plants, peaked at 48 hpi (Fig. 1B). However, contrary to MDA, H_2_O_2_ concentrations were higher in +Si plants than in −Si plants at 24, 48 and 96 hpi by 114.6%, 29.6% and 96.4%, respectively. This indicates that Si amendment functions to increase H_2_O_2_ concentration in plants infested with BPH.

### Responses of antioxidant enzymes to Si amendment and BPH infestation

**Figure 2:**
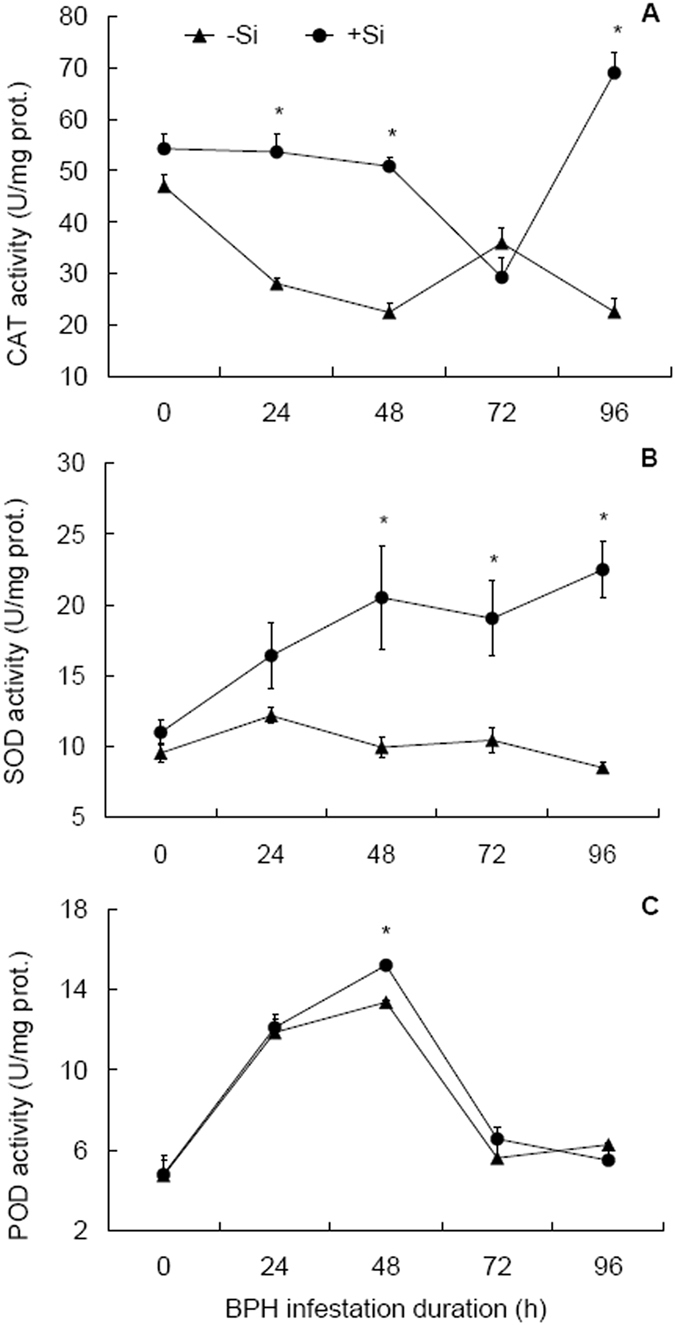
Activities of antioxidant enzymes in rice sheaths in response to Si amendment and BPH infestation. (**A**) Catalase, CAT. (**B**) Superoxide dismutase, SOD. (**C**) Peroxidase, POD. +Si = silicon amendment at 112 mg Si/kg nutrient solution, −Si = no silicon amendment. Error bars represent 1 × SE. *n* = 3 (biological replicates). *Significant difference between + Si and −Si plants at a certain time post BPH infestation at *P* < 0.05 via independent samples T test.

CAT, SOD and POD are antioxidant enzymes involved in scavenge of ROS. Si addition, BPH infestation duration and their interaction all had significant influence on CAT activity (Table 2). With BPH infestation, CAT activities were down-regulated all the way until 72 hpi in +Si plants and until 48 hpi in −Si plants (Fig. 2A). A sharp increase in CAT activity was observed at 96 hpi in +Si plants while a further decrease was evident in −Si plants. Between Si treatments, CAT activities were higher in +Si than in −Si plants at 24, 48 and 96 hpi, by ranges of 91.3%, 126.9%, and 206.4%, respectively, indicating that Si amendment retards the decrease in CAT activity due to BPH infestation.

SOD activities were significantly influenced by Si treatment only (Table 2). With BPH infestation, SOD activities showed a steady increasing pattern in +Si plants while a gradual decreasing pattern in −Si plants (Fig. 2B). Significant differences in SOD activities were observed between +Si and −Si plants at 48, 72 and 96 hpi (by 106.3%, 82.3% and 164.4%, respectively).

Like SOD, Si treatment alone significantly influenced POD activities (Table 2). POD activities were characterized by similar patterns of temporal changes in +Si and −Si plants, showing significant increase (*t* ≥ 6.24, df = 4, *P* ≤ 0.003) at 24 from 0 hpi, peaking at 48 hpi, and then undergoing a significant decrease (*t* ≥ 6.05, df = 4, *P* ≤ 0.004) from 48 to 72 hpi (Fig. 2C). Between the Si treatments, POD activities differed significantly only at 48 hpi, higher in +Si plants than in −Si plants by 18.8%.

### Responses of synthases of secondary metabolites to Si amendment and BPH infestation

**Figure 3:**
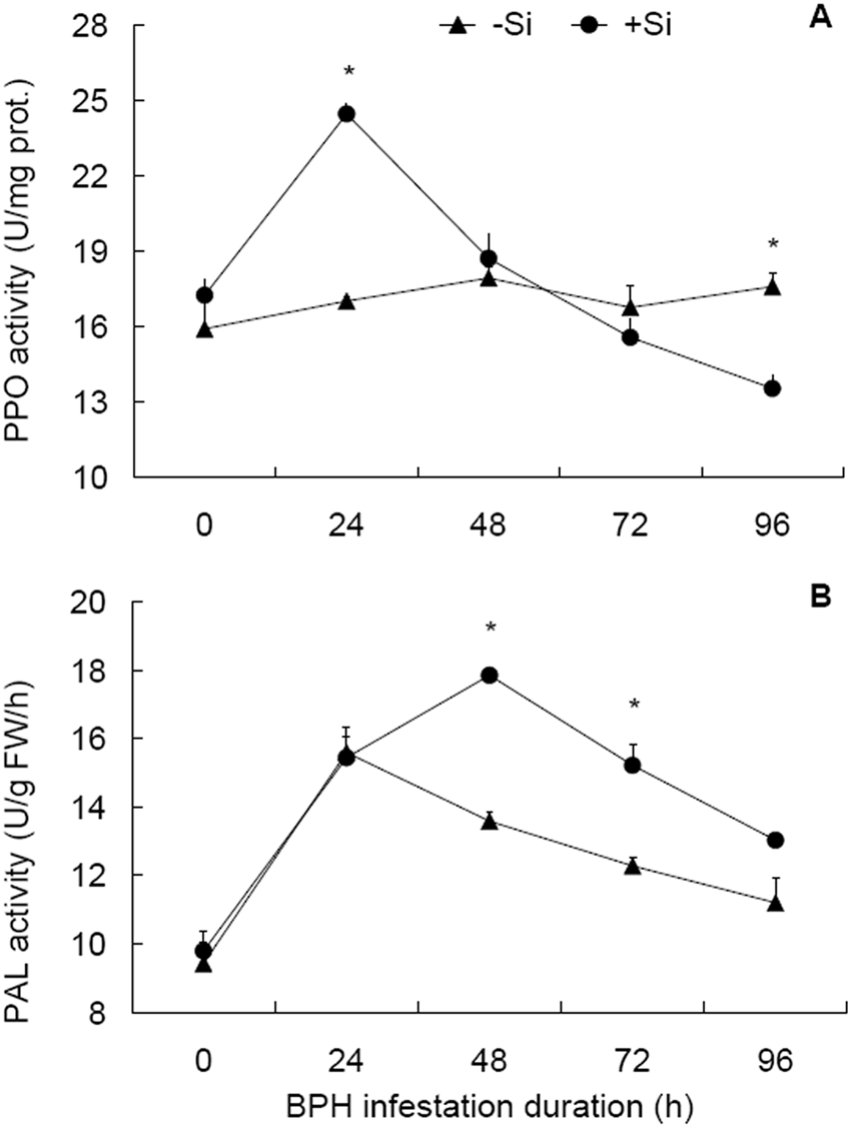
Activities of enzymes for production of secondary metabolites in rice sheaths in response to Si amendment and BPH infestation. (**A**) Polyphenol oxidase, PPO. (**B**) Pheny-lalanine ammonia-lyase, PAL. +Si = silicon amendment to rice plants at 112 mg Si/kg nutrient solution, −Si = no silicon amendment. Error bars represent 1 × SE. *n* = 3 (biological replicates). *Significant difference between +Si and −Si plants at a certain time post BPH infestation at *P* < 0.05 via independent samples T test.

Activities of PPO and PAL, two synthases of secondary metabolites (lignin and phenols), were under significant influence of Si treatment, BPH infestation duration and their interaction (Table 2). PPO activities in +Si plants underwent a significant increase (*t* = 9.423, df = 4, *P* = 0.001) at 24 from 0 hpi and thereafter a significant decrease (*t* = 5.379, df = 4, *P* = 0.006) at 48 hpi (Fig. 3A). In contrast, PPO in −Si plants experienced no significant temporal changes in activity. Between the Si treatments, PPO activities were higher in +Si than −Si plants at 24 hpi and lower in +Si than −Si plants at 96 hpi.

Like POD, PAL activities were also characterized by similar patterns of temporal changes in +Si and −Si plants (Fig. 3B), showing significant increase (*t* ≥ 6.24, df = 4, *P* ≤ 0.003) at 24 from 0 hpi, peaking at 48 hpi in +Si plants and at 24 hpi in −Si plants, and thereafter undergoing significant decrease in +Si plants from 48 to 72 hpi and from 72 to 96 hpi (*t* ≥ 3.586, df = 4, *P* ≤ 0.023) and in −Si plants from 24 to 48 hpi and from 48 to 72 hpi (*t* ≥ 3.57, df = 4, *P* ≤ 0.023). Between the treatments, PAL activities differed significantly at 48 and 72 hpi, higher in +Si plants than in −Si plants by 31.4% and 23.9%, respectively.

### Responses of β-1,3-glucanase to Si amendment and BPH infestation

**Figure 4:**
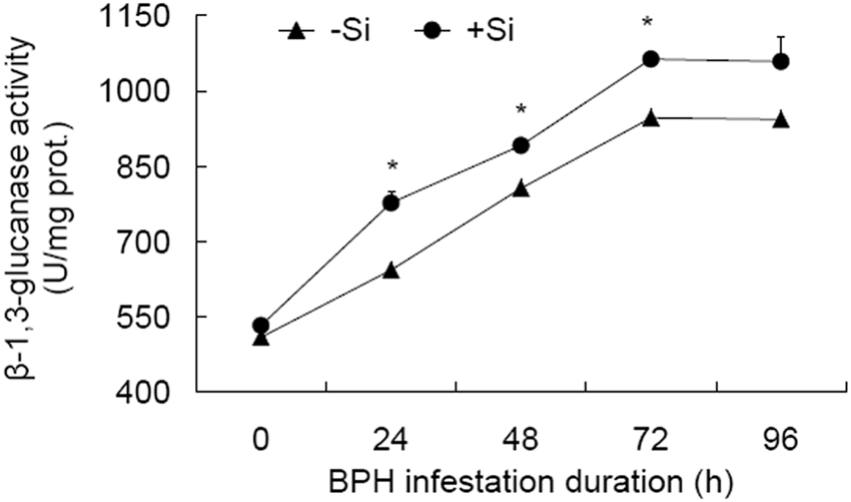
Activitiy of β-1,3-glucanase in rice sheaths in response to Si amendment and BPH infestation. +Si = silicon amendment to rice plants at 112 mg Si/kg nutrient solution, −Si = no silicon amendment. Error bars represent 1 × SE. *n* = 3 (biological replicates). *Significant difference between +Si and −Si plants at a certain time post BPH infestation at *P* < 0.05 via independent samples T test.

β-1,3-glucanase has been extensively reported to be involved in plant defense response towards biotic stress^21^. In this study, both Si treatment and BPH infestation duration exerted significant influence on the activities of β-1,3-glucanase (Table 2). +Si and −Si plants showed similar patterns of temporal changes in the activities of β-1,3-glucanase (Fig. 4), characterized by significant increases from 0 to 24 hpi, from 24 to 48 hpi, and from 48 to 72 hpi (*t* ≥ 4.974, df = 4, *P* ≤ 0.008). Between +Si and −Si plants, β-1,3-glucanase activities were higher at 24, 48 and 72 hpi in the former than in the latter by 20.9%, 10.6% and 13.2%, respectively. These results indicate that β-1,3-glucanase activity responds positively to BPH attack and that Si amendment has somehow enhanced the responses.

## Discussion

Rice is a typical Si hyper-accumulating plant^23^. Although Si is not listed as an essential element for higher plants, its role in mediating resistance to a wide range of abiotic and biotic stresses is beyond doubt^6–9, 18, 24^.

One of the mechanisms of Si-mediated plant resistance to herbivores is that Si is accumulated in epidemic cells, which forms a physical barrier against herbivory^9, 10, 12^. Our present study confirms intensified silicification of rice leaf sheaths (Supplemental Fig. S2, Table 1). This physical barrier mechanism is indicated as one of the key factors for feeding impairment and decreased population growth of BPH on Si-amended rice plants^8^.

However, physical barrier mechanisms are only part of the story of Si’s role in suppressing pests. Si appears to interact with defense-associated signaling pathways and seems to regulate a range of physiological activities in plant stress defenses^5^, one of which is oxidative stress resulting from overproduction of ROS (O^2−^, H_2_O_2_ and OH•) by various biotic and abiotic stresses^15, 25^. ROS are components of plant defense responses to pathogen and herbivore attacks^17^, involved in actin remodeling that is required for defense responses^26^. H_2_O_2_ stimulates a cascade of reactions that lead to the expression of defense genes, preventing the plants from subsequent attack by pathogens and herbivores^27^. Our results showed that H_2_O_2_ contents increased more in +Si than in −Si infested plants (Fig. 1B), which might contribute to the differential expression of defense genes between +Si and −Si plants. H_2_O_2_ may also have potential for providing resistance to herbivores through a direct effect on insect physiology, as in the case of the European corn borer (*Ostrinia nubilalis*)^28^. Therefore, it can be reasoned that the increased H_2_O_2_ content in the infested +Si plants_,_ might partially explain the poor population growth recorded for BPH on Si-amended rice plants^8^. High levels of lipid peroxidation caused by ROS can results in increased MDA, which is used as a biomarker of the degree of cell membrane damage^15^. It was found that MDA contents in leaf sheaths generally increased in response to BPH infestation, but the increases were remarkable in −Si plants in contrast to +Si plants (Fig. 1A). Similar results of Si addition in reducing MDA concentration is reported in the interaction between rice plants and leaf folder, *Cnaphalocrocis medinalis* ^12^. These results indicate that Si amendment functions to reduce MDA accumulation in plants attacked by herbivores, and thus provide protection of the stressed plants.

ROS at excessive levels can cause significant damage to cell structures^25^, plants protect themselves from excessive levels of ROS with antioxidant enzymes (SOD, POD and CAT)^17^. SOD removes superoxide anion free radicals accompanying the formation of H_2_O_2_, which is then detoxified by POD and CAT^29^. In the present study, the activities of SOD and POD generally increased and that of CAT was down-regulated in response to BPH infestation; but in +Si plants, CAT, SOD and POD activities were higher at certain time post-infestation than in −Si plants (Fig. 2). Similar results were reported by Han *et al*.^12^ in Si-amended rice plants’ responses to infestation of *C. medinalis* and in Si-amended *Arabidopsis* ^30^, rice^31^ and perennial ryegrass^32^ plants’ responses to disease infection. These results indicate that Si plays a role in removing excessive levels of ROS by priming activities of antioxidant enzymes.

Secondary metabolic compounds are also key components in plant resistance to biotic stress^5, 33^. PAL is involved in the biosynthesis of phenolics, phytoalexins and lignins; PPO catalyzes oxidation of phenols to quinines that can restrict development of herbivorous insects^34, 35^. Our results revealed that PPO and PAL activities were triggered right after BPH infestation and thereafter, showed a declining pattern in both +Si and −Si plants, but they were generally high in +Si plants in contrast to −Si plants (Fig. 3). In the defense responses of wheat plants to infestation by *Schizaphis graminum* ^33^ and rice plants to infestation by *C. medinalis* ^12^, Si triggers the activities of PPO and PAL and confers increased resistance to herbivores. Although further investigation is needed to test whether increases in PPO and PAL activities have resulted in increased contents of the secondary metabolic compounds, it can be reckoned that Si amendment, through priming of synthases of secondary metabolites, contributes to the reduced population growth in BPH on Si-amended rice plants. However, a recent report has demonstrated that Si-amended sugar plants have lower concentrations of phenolic compounds in roots while afford enhanced resistance to root herbivores^20^, indicating a possible trade-off between carbon-based defense compounds, such as phenolics, and silicon-based defenses, especially in plant roots.

β-1,3-glucanase, acting alone and particularly in combination with chitinase, contributes to plant defense^21^. BPH infestation activated β-1,3-glucanase in the current study, and this activation was greater in +Si plants than in −Si plants (Fig. 4). Similarly, Alagar *et al*.^22^ noticed a higher activity of β-1,3-glucanase in resistant cultivars than in susceptible genotypes of rice attacked by BPH. The increased activities of β-1,3-glucanase protected potato plants against the disease caused by *Rhizoctonia solani* AG-3^36^. Therefore it is evident that the enhanced activities of β-1,3-glucanase associated with Si amendment may have benefited Si-mediated rice plants in their resistance to BPH attack.

In summary, our results show that Si amendment significantly alters activities of antioxidant enzymes, synthases for secondary metabolites, and β-1,3-glucanase that are induced by BPH infestation. Our findings demonstrate that Si amendment, through interactions with BPH infestation, plays a role in priming intensified plant defense responses that finally lead to enhanced plant resistance to BPH. These results, along with findings from previous investigations^5, 12, 33^, have furthered our understanding of the role of Si from a physical barrier to priming of defense responses for enhanced plant resistance to herbivores. In the circumstances of high insecticide resistance in BPH^2^, severe damage caused by BPH, and no commercial rice varieties resistant to BPH available^1^, our findings point to a potential alternative for BPH management that is of important agricultural and ecological implications.

## Methods

### Rice plants and Si treatment

Rice plants of a susceptible variety Taichuong Native 1 (TN1) was used both to rear the brown planthopper and as test plants. After germination, the seeds were sown in stainless steel plates (27 × 19 × 4 cm) with sands and tap water. Ten-day old seedlings were transplanted to plastic boxes (50 × 40 × 15 cm) at 20 plants per box, where the plants were aquacultured with nutrient solution (about 12,000 ml/box) according to Yoshida *et al*.^37^. The nutrient solution was prepared using deironized water and controlled for pH of 5.0–6.0. Si amendment (+Si) was established since transplanting by adding Na_2_SiO_3_·9H_2_O to the nutrient solution at 112 mg Si/L, a control without addition of Na_2_SiO_3_·9H_2_O (−Si) was included. The nutrient solution was replenished every 5 d. The plants were cultured in a greenhouse to prevent rain and natural occurring pests.

### Planthoppers

A stock culture of the brown planthopper was maintained in greenhouse cages (40 × 40 × 40 cm) with 30–45-d old potted TN1 seedlings. To obtain experimental planthopper populations, adults were periodically transferred from the greenhouse cages to cages with 20-d old rice seedlings in climate chambers (26 ± 1 °C, RH 85% ± 5%, 14 L:10D) for oviposition. After 24 h, the seedlings were replaced and the seedlings with eggs were cultured in insect-free cages in the chambers until the nymphs therein reached the 5th stadium when they were transferred to glass tubes (2.5 × 15 cm) with aquacultured rice seedlings. Newly emerged macropterous female adults were used in the experiments. The plants used for insect rearing were not amended with Si.

### Determination of Si content and microscopic observation of silica cells

Si content was measured to samples of both +Si and −Si rice plants infested with BPH or not. Six boxes of +Si plants (each with 20 plants) were divided into two groups: one was exposed to 200 newly emerged BPH females in cage while the other was not. Another six boxes of −Si plants were treated in the same way. Rice stems 12 cm above the first node were harvested at 96 hpi or from uninfected plants and flushed with tap water to get rid of any mud, and then were killed at 110 °C for 15 min and dried at 80 °C to constant weight. Each box of plants served as a replication, three boxes of plants were sampled. The dried leaf sheaths from a replicate were crushed with a food pulverizer and sieved with a 0.245-mm screen. Si contents were measured from the resulted leaf sheath powder using the procedures of Dai *et al*.^38^.

Silica cells on leaf sheath surface were observed morphologically using a scanning electron microscopy (SEM) (Hitachi S-570, Japan). Fresh leaf sheath samples were obtained from +Si and −Si rice plants infested for 96 h with BPH or not. Specimens for SEM observation were prepared as described by Han *et al*.^12^. Fifteen SEM pictures (at 100 × magnification) were randomly selected from each treatment to determine rows of silica cells per 1 mm^2^ area and numbers of silica cells per 1-mm row. The length, width and area of silica cells were measured by Image-Pro Plus (Version 6.0, Georgia, USA) from 100 silica cells for each treatment. In addition, SEM pictures (at 300 × magnification) were obtained to show the silica cells.

### Sampling of planthopper-infested plants

To test for the effects of Si amendment and BPH infestation on defense responses in rice plants, one aquacultured 30-d old (i.e. 20 d after transplanting) rice seedling, either +Si or −Si, was exposed to 10 macropterous female adults in a glass tube in a climatic chamber. A sponge disc (3 cm in diameter and 2 cm thick) was used to secure the seedling at 6 cm above roots and another sponge disc was used to seal the tube opening, thus leaving a space of 4-cm height in the tube, where the insects were transferred. Nutrient solution was added to the bottom of the tube. The 4-cm segment of the rice stem was sampled at 0, 24, 48, 72, or 96 h post-infestation (hpi) of BPH using a clean scissor and the leaf sheaths were collected into a valve bag and then kept at −80 °C. For each combination of Si treatment and BPH infestation duation, sampling was repeated 3 times from different rice seedlings. The leaf sheath samples thus collected were used to measure the activities of antioxidant enzymes (CAT, POD and SOD), synthases of secondary metabolites (PPO and PAL) and β-1,3-glucanase. They were also used to determine the concentrations of MDA and H_2_O_2_.

### Preparation of samples for chemical analysis

Leaf sheaths (ca. 0.3 g) were ground thoroughly in a mortar together with liquid nitrogen and phosphate buffer solution (pH = 7.4) at 1:9 (weight:volume), and then the mixture was homogenized in iced water. The homogenate was centrifuged (5417 R, Eppendorf, Hamburg, Germany) for 15 min at 2,500 r/min at 4 °C. The supernatant was used in determination of activities of CAT, SOD, POD, PPO, or PAL, and concentrations of MDA or H_2_O_2_. For each measurement of the parameters, separate supernatant samples were prepared.

For measurement of β-1,3-glucanase activity, leaf sheaths (ca. 0.3 g) ground thoroughly in a mortar together with liquid nitrogen were mixed with 0.1 mol/L sodium citrate buffer solution (pH = 5.0) at 1:9 (weight:volume). After the mixture was maintained in ice box for 1 h, it was centrifuged for 20 min at 12,000 r/min at 4 °C. The supernatant was used in measurement of β-1,3-glucanase activity.

### Measurement of concentration of MDA and H_2_O_2_

MDA is the most abundant aldehydic lipid breakdown product that indicates the levels of stress and injury to the functional membrane^15^. MDA content was determined by the thiobarbituric acid method^39^ using an MDA assay kit (Nanjing Jiancheng Bioengineering Institute, Nanjing, China) and a Multiskan Spectrum (Thermo Fisher Scientific Ltd., Finland). H_2_O_2_ content was determined by the ammonium molybdate spectrophotometric method^40^. All these measurements were biologically repeated for 3 times.

### Tests for enzyme activities

The methods of tests for enzyme activities were largely the same as reported by Han *et al*.^12^. Activities of antioxidant enzymes (CAT, POD and SOD) and synthases (PPO and PAL) of secondary metabolite in the rice leaf sheaths were tested using respective diagnostic kits (Nanjing Jiancheng Bioengineering Institute, Nanjing, China). CAT activity was measured according to the ammonium molybdate spectrophotometric method^41^. POD activity was determined by a spectrophotometer (UV-2000, UNICO, Shanghai, China) following the change of absorption at 420 nm due to guaiacol oxidation^42^. For determination of SOD activity, the 2-(4-iodophenyl)-3-(4-nitrophenyl)-5-(2,4-disulfophenyl)-2H-tetra- zolium (WST-1) method was used^43^. Activities of PPO and PAL were assayed according to the methods of Cai *et al*.^31^. Activity of β-1,3-glucanase was measured according to Alagar *et al*.^22^. The tests were biologically repeated for 3 times.

### Data analysis

All data in figures and tables are shown as means ± SE. The data were subjected to two-way analysis of variance (ANOVA) for the effects of Si amendment, BPH infestation duration and the interactions between the two factors. The means were separated by Tukey’s multiple range test or by independent-samples t-test (*P* = 0.05) for significant differences between treatments. All statistical analysis was performed using SPSS 16.0 (SPSS Inc, USA).

## Electronic supplementary material

supplementary information file
