# Supplementary material for: Silicon amendment is involved in the induction of plant defense responses to a phloem feeder
Source: Sci Rep. 2017 Jun 26;7:4232. doi: 10.1038/s41598-017-04571-2 (PMC5484686; doi:10.1038/s41598-017-04571-2)
Supplement: Supplementary file 1 — supplementary information file [file 41598_2017_4571_MOESM1_ESM.pdf]

Silicon amendment is involved in the induction of plant defense responses to a phloem feeder

Lang Yang[1,2], Yongqiang Han[3], Pei Li[1,2], Fei Li[1,2], Shahbaz Ali[1,2], and Maolin Hou[1,2,*]

[1] State Key Laboratory for Biology of Plant Diseases and Insect Pests, Institute of Plant Protection, Chinese Academy of Agricultural Sciences, Beijing 100193, China

[2] Southern Regional Collaborative Innovation Center for Grain and Oil Crops in China, Changsha 410128, China

[3] Institute of Plant Protection of Hunan Province, Changsha 410125, China

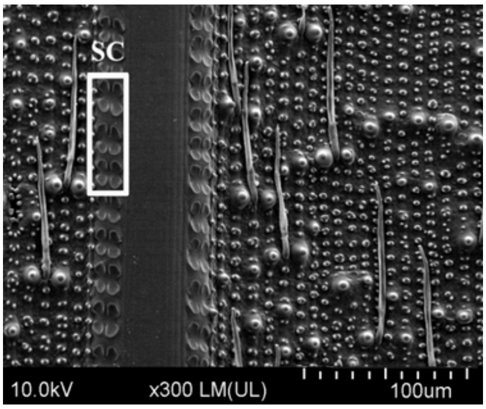

Supplemental Figure S1. Scanning electron micrographs (300 ×) of rice sheath surface showing silica cells (SC) along the vein.

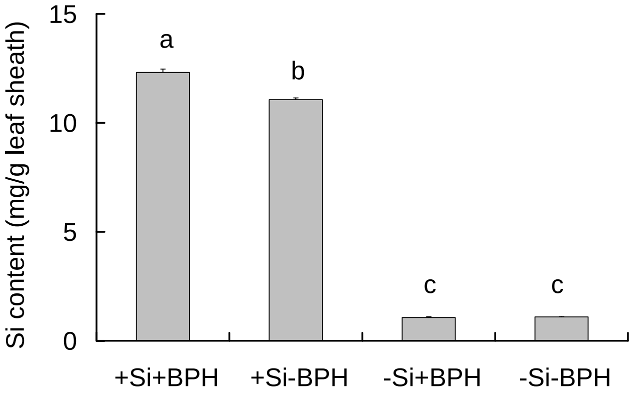

Supplemental Figure S2. Effect of Si amendment and BPH infestation on Si content. +Si = Si amendment to rice plants at 112 mg Si/kg nutrient solution, –Si = no Si amendment. Error bars represent 1 × SE. n = 3 (biological replicates). Different letters over the bars denote significant difference at *P* < 0.05 via Turkey's multiple range tests.
